# Use of Wearable Sensors and Biometric Variables in an Artificial Pancreas System

**DOI:** 10.3390/s17030532

**Published:** 2017-03-07

**Authors:** Kamuran Turksoy, Colleen Monforti, Minsun Park, Garett Griffith, Laurie Quinn, Ali Cinar

**Affiliations:** 1 Department of Biomedical Engineering, Illinois Institute of Technology, 3255 S. Dearborn St., Chicago, IL 60616, USA; kturksoy@iit.edu; 2 Department of Chemical and Biological Engineering, Illinois Institute of Technology, 10 W. 33rd St., Chicago, IL 60616, USA; cmonfort@hawk.iit.edu; 3 College of Nursing, University of Illinois at Chicago, 845 S. Damen Ave., MC 802, Chicago, IL 60612, USA; mpark61@uic.edu (M.P.); lquinn1@uic.edu (L.Q.); 4 Integrative Physiology Laboratory, University of Illinois at Chicago, 1640 W. Roosevelt Rd., Chicago, IL 60612, USA; gjgriff@uic.edu

**Keywords:** artificial pancreas, wearable sensors, biometric variables, exercise, type 1 diabetes, partial least squares

## Abstract

An artificial pancreas (AP) computes the optimal insulin dose to be infused through an insulin pump in people with Type 1 Diabetes (T1D) based on information received from a continuous glucose monitoring (CGM) sensor. It has been recognized that exercise is a major challenge in the development of an AP system. The use of biometric physiological variables in an AP system may be beneficial for prevention of exercise-induced challenges and better glucose regulation. The goal of the present study is to find a correlation between biometric variables such as heart rate (HR), heat flux (HF), skin temperature (ST), near-body temperature (NBT), galvanic skin response (GSR), and energy expenditure (EE), 2D acceleration-mean of absolute difference (MAD) and changes in glucose concentrations during exercise via partial least squares (PLS) regression and variable importance in projection (VIP) in order to determine which variables would be most useful to include in a future artificial pancreas. PLS and VIP analyses were performed on data sets that included seven different types of exercises. Data were collected from 26 clinical experiments. Clinical results indicate ST to be the most consistently important (important for six out of seven tested exercises) variable over all different exercises tested. EE and HR are also found to be important variables over several types of exercise. We also found that the importance of GSR and NBT observed in our experiments might be related to stress and the effect of changes in environmental temperature on glucose concentrations. The use of the biometric measurements in an AP system may provide better control of glucose concentration.

## 1. Introduction

Diabetes mellitus is a chronic metabolic disease in which people cannot properly regulate their blood glucose levels due to inadequate insulin production and/or insulin resistance. This leads to hyperglycemia (high blood glucose concentration) because insulin, a hormone produced by the pancreas, is deficient or ineffective in reducing the blood glucose concentration (BGC). Type 1 diabetes (T1D) is a chronic autoimmune disease that can occur at any age, but it is most often diagnosed in children, teens, or young adults. In T1D, the body produces only a negligible amount of insulin; therefore, patients require daily administration of exogenous insulin to survive. The exact cause is unknown, but it is likely that it results from interplay among autoimmunity, genetics, and environment.

People with T1D use one of two main approaches to deliver exogenous insulin: insulin injections or insulin pump therapy. Insulin injections are usually administered subcutaneously in 3–5 doses daily; insulin pumps are used to infuse a programmed basal dose of insulin subcutaneously 24/7 with bolus doses before meals. The goal of either approach is to maintain BGCs in the optimal range (70–180 mg/dL). An artificial pancreas (AP) automates insulin pumps by using a closed-loop controller that receives information from sensors, computes the optimal insulin amount to be infused, and manipulates the infusion rate of the pump.

It has been recognized that exercise provides a major challenge in the development of closed-loop AP systems for people with T1D, however, the physiology and effects of this perturbation are not very well understood. It is known that exercise induces hypoglycemia in T1D due to increased glucose uptake as well as inadequate glucagon secretion and/or hepatic glucagon sensitivity [1]. However, different types (e.g., aerobic or resistance), duration, and intensity of exercise influence the development of hypoglycemia. The lack of physiological models of insulin action and glucose uptake during exercise prevents quantification of the effect of exercise on insulin sensitivity as well as other variables, especially in T1D. 

The use of biometric physiological variables in an AP system may be beneficial for prevention of exercise-induced challenges and better glucose regulation in people with T1D [2]. Few studies have been done to investigate the effects of biometric variables in AP systems that may be used to improve performance and mitigate exercise-induced hypoglycemia.

Dasanayake et al. [3] developed a method to detect the start and end of exercise in people with T1D before any significant change in BGC occurred. Outpatient ambulatory data from an accelerometer, heart rate monitor (HRM), continuous glucose monitor (CGM), insulin pump, and glucose meters were collected from sixteen subjects over the first two study days to inform the detection algorithm. On the third study day subjects performed aerobic exercise for 1 h at 30% predicted maximal heart rate reserve (HRR) in the morning and for 30 min at 50% HRR in the afternoon. Subjects were then discharged and collected data for two more days. Principal component analysis (PCA) was used for activity detection due to the high correlation between heart rate (HR) and acceleration data. The study concluded that this detection method identified the onset and end of exercise in approximately 5 min, with an average BGC change of −6 mg/dL. 

In the first study done by Stenerson et al. [4] researchers also began by collecting free-living data from subjects with T1D using a combined accelerometer and HRM, an insulin pump, and a CGM in order to supplement an existing predictive low glucose suspend (PLGS) algorithm. Different algorithms were tested in a computer simulation and compared based on reduction in exercise-related hypoglycemia. They found that the PLGS algorithm reduced hypoglycemia by 62%, while the HRM-augmented algorithm and the accelerometer-augmented algorithm reduced hypoglycemia by 71% and 74%, respectfully. Combined HRM and accelerometer augmentation reduced hypoglycemia by 76%. In a follow-up study, researchers compared the effectiveness of the accelerometer-augmented PLGS algorithm with subjects’ regular basal insulin rate during an outpatient exercise protocol [5]. Eighteen subjects participated in an on-algorithm soccer session and an off-algorithm soccer session where the rate of hypoglycemia during and after exercise was compared. It was found that the difference in meter BGC levels between groups did not achieve statistical significance at any time. Additionally, the accelerometer-augmented algorithm failed to prevent hypoglycemia when compared to subjects using their usual basal rates. However, researchers suggested that the small sample size may have contributed to the lack of statistical significance.

Breton et al. [6] compared the success of a control-to-range (CTR) AP system with a CTR system informed by heart rate (CTR + HR) based on the following metrics: BGC decline during exercise, the Low Blood Glucose Index (LBGI), number of hypoglycemic episodes (BG < 70 mg/dL), and overall glucose control (% time with BG between 70 and 180 mg/dL). Twelve subjects participated in two 26-h sessions including 30 min of mild exercise. The CTR + HR system significantly reduced the BGC decline during exercise; marginally reduced LBGI; allowed fewer hypoglycemic events during exercise; and increased the amount of time within the target BGC range. LBGI and average BGC remained constant. Despite the small sample size, this study did find a statistically significant result in that the BGC rate of decline during exercise was reduced when HR information was supplied to the closed-loop controller. However, the subjects in this study had no cardiovascular complications and performed only mild exercise. Additionally, the HR signal collection was triggered manually rather than starting based on prediction of exercise.

In the first study done by Cichosz et al. [7] researchers gathered CGM data and heart rate variability (HRV) data from ten subjects with T1D while bedridden during insulin-induced hypoglycemia. These data were used to develop an algorithm that detected hypoglycemic periods using pattern recognition to identify when the BGC level was below 3.9 mmol/L. For a ten-minute prediction, the algorithm provided 79% sensitivity (true positive hypoglycemia rate) and 99% specificity (true negative hypoglycemia rate) for a total of 903 samples, detecting 16/16 hypoglycemic events with no false positives and a 22-min lead time compared to the CGM alone. In a follow-up study, researchers used this pattern-classification algorithm to predict hypoglycemia based on three different models: (i) a model containing raw CGM data; (ii) a model containing data derived from CGM signal; and (iii) a model containing data derived from CGM and HRV signals [8]. The algorithm was previously tested in a hospital setting where the subjects were bedridden and subjected to induced hypoglycemia, but in this study the twenty-one subjects were monitored while performing normal daily activities in order to predict spontaneous hypoglycemia. For a 20-min prediction, model (i) resulted in a receiver operation conditions (ROC) area under curve (AUC) of 0.69 and had a 69% specificity for a 100% sensitivity, model (ii) had a ROC AUC of 0.92 with a 71% specificity for a 100% sensitivity, and model (iii) had a ROC AUC of 0.96 with a 91% specificity for a 100% sensitivity. Combining CGM and HRV data improved hypoglycemic prediction.

Our group previously reported results for an adaptive control system using CGM measurements as well as energy expenditure and galvanic skin response to predict future glucose concentrations without meal or activity announcements [9]. Insulin-on-board (IOB) was added starting with the third experiment, and the algorithm was modified before each new experiment based on its performance in previous experiments in order to improve performance. The control algorithm was tested during seven closed-loop experiments 32 or 60 h in length with three different patients with T1D. This control system kept glucose concentration in the normal range (70–180 mg/dL) for 62% of the closed-loop experiment observations. Mild hypoglycemic episodes occurred in the final experiment after IOB was added. In a subsequent study [10], we integrated a hypoglycemia early alarm (HEA) system with the multivariable adaptive artificial pancreas (MAAP) control system. This integrated MAAP (IMAAP) system used continuous glucose measurements, energy expenditure and galvanic skin response, and sleep information in order to regulate blood glucose during closed-loop experiments. The HEA system was able to predict hypoglycemic episodes an average of 25 min before they occurred. Eleven open-loop experiments and nine closed-loop experiments, including three with the IMAAP system, were conducted. Severe hypoglycemia occurred during open-loop experiments, but the MAAP system reduced the incidence and severity of hypoglycemia significantly (*p* < 0.01), and with the IMAAP system, hypoglycemia was avoided completely despite two unannounced exercise sessions per day during three-day long closed-loop experiments. 

It has been illustrated in many studies that inclusion of biometric variables in AP control systems improves performance by reducing the incidence of hypoglycemia and increasing time within the normal range of glucose concentration [11]. The goal of the present study is to find a correlation between biometric variables and changes in glucose concentration via partial least squares (PLS) regression and variable importance in projection (VIP) using open-loop data in order to determine which variables would be most useful to include in a future artificial pancreas control system to mitigate the effects of exercise on blood glucose variability [12].

**Figure 1:**
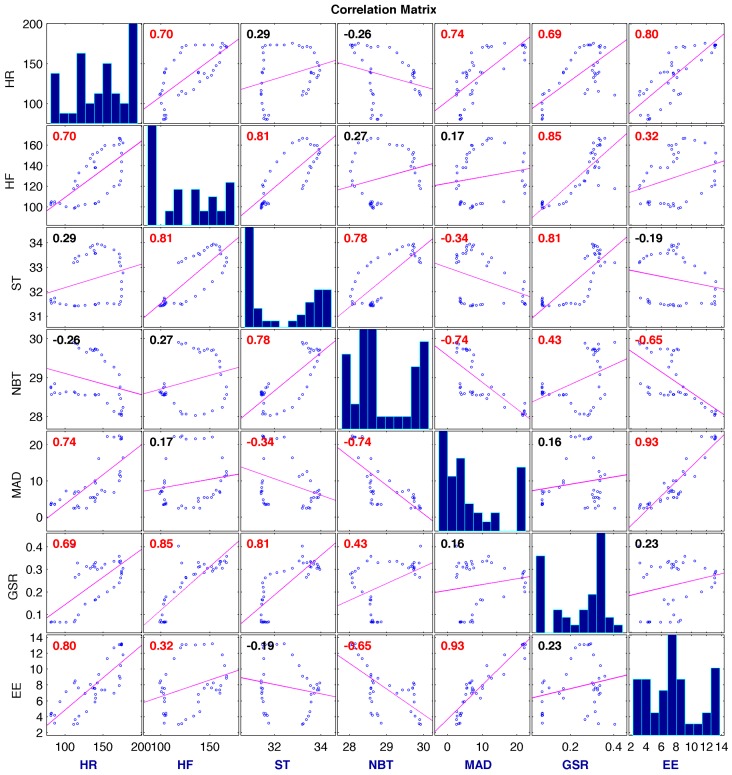
Linear regressions between biometric variables for 1 treadmill exercise session. Red colored correlation coefficients indicate statistical correlation. The diagonal figures show histograms for each biometric variable.

PLS regression is an effective method for developing an empirical model in cases with multicollinearity as well as when there is a larger number of predictor variables than the number of samples. Both of these limiting factors are present in this clinical data set, because biometric variables are highly correlated and there are significantly more predictor variables than samples for each exercise period in the experiment (See Figure 1 and Results section). 

PLS has also been used across a variety of disciplines in order to determine correlation in these limiting cases. PLS has been used to investigate the relations between different measurements in different areas. Variable Importance in Projection (VIP) metric was used to quantify the contribution of each predictor variable to the PLS components [13,14,15,16]. This broad application and success of PLS regression techniques provides evidence that it can be used to find a correlation between biometric variables and glucose variability in order to improve future AP control systems.

## 2. Materials and Methods

### 2.1. Partial Least Squares (PLS)

PLS is a powerful linear regression method due to its ability to accurately model data for instances of multicollinearity as well as in situations where the number of predictor variables is much larger than the number of samples. The PLS model can be expressed as shown in Equations (1) and (2) below:

(1) $$X=TP^{T}+E=\sum_{i=1}^{h}t_{i}p_{i}^{T}+E$$

(2) $$Y=UQ^{T}+F=\sum_{i=1}^{h}u_{i}q_{i}^{T}+F$$

where ***X*** is the matrix of predictor variables, ***Y*** is the response variable matrix, ***T*** is the matrix of ***X***-scores, ***U*** is the vector of ***Y***-scores, ***P*** is the matrix of ***X***-loadings, ***Q*** is the weight matrix for ***Y***, ***E*** is the random error of ***X*** and ***F*** is the random error of ***Y***. PLS selects latent variables so that the variation in ***X*** that best explains the variation in ***Y*** is recovered. This selection allows the size of the data set to be reduced while maintaining as much predictive power as possible.

In order to maximize the covariance in ***X*** and ***Y*** explained by as few variables as possible, linear combinations of predictor vectors are calculated from latent variable scores *t_i_ = w_i_^T^x* and response variable scores *u_i_ = q_i_^T^y*, where *w* and *q* are the weight vectors for *x* and *y*.

For the first latent variable:

(3) $$w_{1}^{T}=\frac{u_{1}^{T}X}{∥u_{1}^{T}u_{1}∥},\;t_{1}=\frac{Xw_{1}}{∥w_{1}^{T}w_{1}∥}$$

(4) $$q_{1}^{T}=\frac{t_{1}^{T}Y}{∥t_{1}^{T}t_{1}∥},\;u_{1}=\frac{Yq_{1}}{∥q_{1}^{T}q_{1}∥}$$

Convergence occurs when *t*_1_ is within a certain threshold of *t*_1_ from an earlier iteration. After convergence, ***X***-loadings are calculated and the scores and weights are adjusted:

(5) $$p_{1}^{T}=\frac{t_{1}^{T}X}{∥t_{1}^{T}t_{1}∥},\;p_{1n}=\frac{p_{1o}}{∥p_{1o}∥}$$

(6) $$t_{1n}=t_{1o}∥p_{1o}∥,\;w_{1n}=w_{1o}∥p_{1o}∥$$

where *o* is old and *n* is new. The regression coefficient is calculated using Equation (6) below:

(7) $$b_{1}=\frac{t_{1}^{T}u_{1}}{∥t_{1}^{T}u_{1}∥}$$

In order to exclude the variance explained by the first latent variable from additional latent variables, ***X*** and ***Y*** are replaced by their residuals in the next iteration.

### 2.2. Variable Importance in Projection (VIP)

The variable importance in projection method of variable selection in PLS (PLS-VIP) scores the importance of the *j*-th variable based on Equation (8) below, where *p* is the number of predictor variables, and *h* is the number of latent variables:

(8) $$VIP_{j}=\sqrt{\frac{p\sum_{k=1}^{h}\left(t_{k}^{T}t_{k}\right)\left(q_{k}^{T}q_{k}\right)w_{jk}^{2}}{\sum_{k=1}^{h}\left(t_{k}^{T}t_{k}\right)\left(q_{k}^{T}q_{k}\right)}}$$

The PLS-VIP measures the contribution of each predictor variable to the model. The average of all squared PLS-VIP scores for one PLS model is equal to 1, so any predictor variable that has a VIP score greater than or equal to 1 is considered important. Any predictor variable with a VIP score less than 0.5 is considered unimportant, and any predictor variable with 1 > VIP ≥ 0.5 is considered marginally important. An important variable contributes useful information to the model, while unimportant variables do not. Conclusions cannot be reached about the marginally important variables without further analysis. 

### 2.3. Experimental Setup

Overall 26 subjects with T1D participated in the clinical experiments. Table 1 shows the characteristics of the participants.

The experiments were conducted under two different protocols. The details of the protocols are given in the next section. Several types of exercises were tested. Table 2 shows the types of exercise that were tested on each subject along with subjects’ demographic information.

#### 2.3.1. Protocol 1

The subjects met at the University of Illinois-College of Nursing (UIC-CON) where the study was explained and informed consent was obtained. A brief health history and physical examination was performed; hemoglobin A1C (A1C) (A1CNow+; Bayer, Metrika, Sunnyvale, CA, USA) was obtained; and subjects completed the Hypoglycemia Fear Survey II (HFS) [17]; the Diabetes Treatment Satisfaction Questionnaire (DTSQ) [18]; Hospital Anxiety and Depression (HAD) Scale [19]; and the Illness Intrusion Ratings Scale [20]. A SenseWear^®^ physical activity monitor (Body Media, Pittsburgh, PA, USA) was attached to the non-dominant arm of each subject. Continuous Glucose Monitors (Guardian-Real Time Continuous Glucose Monitors, Medtronic, Northridge, CA, USA; or US G4 PLATINUM (DEXCOM, San Diego, CA, USA) were attached to the abdominal area or other area of subject preference (e.g., upper arm). The subjects were instructed to document their diet and physical activity throughout the remainder of the study period. Subjects wore the CGM and physical activity devices for a period of 6 days. The devices were downloaded at the next study appointment.

The subjects participated in 3–4 visits to the exercise laboratory. The duration of each visit was 4–8 h and the subjects wore CGM, physical activity, and HR (Mio Alpha, MIO Global, Vancouver, BC, Canada) monitors continuously. The following tests and procedures (described below) were performed during these visits: peak oxygen consumption (VO2MAX) test; and submaximal aerobic exercise bouts. Additionally, a dual energy absorptiometry (DEXA) scan was performed. The subjects spent the majority of time in the exercise laboratory where they alternately rested and performed submaximal aerobic exercise bouts. The subjects ate 1–2 meals and snacks while in the laboratory, as appropriate. The CGM, physical activity, and HR data were downloaded periodically throughout the study.

#### 2.3.2. Protocol 2

The subjects met at the UIC-CON where the study was explained and informed consent was obtained. A brief health history and physical examination was performed; hemoglobin A1C (A1C) (A1CNow+) was obtained; and subjects completed the Hypoglycemia Fear Survey II (HFS) [17]; the Diabetes Treatment Satisfaction Questionnaire (DTSQ) [18]; Hospital Anxiety and Depression (HAD) Scale [19]; and the Illness Intrusion Ratings Scale [20]. A SenseWear^®^ physical activity monitor (Body Media, Pittsburgh, PA, USA) was attached to the non-dominant arm of each subject. A continuous glucose monitor (CGM, DEXCOM US G4 PLATINUM) was attached to the abdominal area. The subjects were instructed to document their diet and physical activity throughout the remainder of the study period. Subjects wore the physical activity and CGM devices for a period of 6 days. The devices were downloaded at the next study appointment.

The subjects participated in 4–5 visits to the exercise laboratory. The duration of each visit was 4–8 h and the subjects wore CGM, physical activity, HR (Mio Alpha) and physiological monitors (BioHarness 3, Zephyr Technology, Annapolis, MD, USA). The following tests and procedures (described below) were performed during these visits: peak oxygen consumption (VO2MAX) test; muscle strength tests; a submaximal resistance exercise bout; and submaximal aerobic exercise bouts. The subjects spent the majority of time in the exercise laboratory where they alternately rested and performed exercise bouts. The subjects ate 1–2 meals and snacks while in the laboratory, as appropriate. The CGM, physical activity, HR and physiological data were downloaded periodically throughout the study.

#### 2.3.3. Aerobic Capacity

Aerobic capacity, or peak oxygen consumption (VO2MAX), was assessed using the Bruce Protocol [21] and an open-circuit spirometry metabolic cart system (True One, Parvo Medics, Sandy, UT, USA) for measurement of expired gases. Subjects were monitored continuously with a full 12-lead ECG before, during, and after the graded exercise test. The subjects performed a 1-min warm-up at 1.7 miles per hour and no incline prior to initiation of the test protocol. Tests were completed upon volitional fatigue, and VO2MAX was expressed as mL∙kg^−1^∙min^−1^ based on the highest recorded 30 s value when two of the following three conditions were satisfied: (1) respiratory exchange ratio (RER) ≥ 1.10; (2) maximum HR within 10 beats∙min^−1^ of age-predicted maximum (220-age); or (3) peak rating of perceived exertion (RPE) ≥ 17.

#### 2.3.4. Muscular Strength

Subjects completed a 5-min warm-up consisting of treadmill walking at a self-selected pace. Muscular strength was assessed using a standardized one repetition maximum (1RM) protocol for six exercises: (1) dumbbell chest press; (2) lat pull down; (3) seated row; (4) dumbbell shoulder press; (5) leg extension; and (6) leg curl. A rest period of 3 min was allowed between each set for each exercise. Subjects performed a warm-up set of 10 repetitions for a given exercise at estimated 50% of 1RM, followed by a set of five repetitions at estimated 70% of 1RM, and a set of three repetitions at estimated 80% of 1RM. Subjects then incrementally increased the weight after each successful 1RM attempt, defined as controlling the movement through the entire concentric and eccentric motions. 1RM was determined to be the most weight lifted one time after three consecutive failures of an increased weight.

#### 2.3.5. Submaximal Resistance Exercise Bout

The submaximal resistance training bout took place ≥7 days after the 1RM protocol. Subjects completed a 5-min warm-up consisting of treadmill walking at a self-selected pace. Subjects performed the same six exercises that were completed during 1RM testing. The resistance training bout consisted of two sets with eight repetitions each at 60% of 1RM. Rest periods of 3 min were taken between each exercise, and subjects completed an entire circuit of all six exercises before beginning the second set of each.

#### 2.3.6. Submaximal Aerobic Exercise Bout

The subjects participated in 30–40 min of submaximal aerobic exercise using the treadmill and bicycle ergometer. The speed and incline of the treadmill were increased until the subjects met a pre-determined HR based on their 60%–90% of their VO2MAX or predicted HR [22]. The speed and load of the bicycle ergometer were increased until the subjects met a pre-determined HR based on their 60%–90% VO2MAX or predicted HR. The range of exercise intensity reflects intervals designed to alternate between moderate and high-intensity aerobic exercise.

## 3. Results

The predictor variables (***X***) used in this study are AUC of heart rate (HR, BPM) from the Mio Alpha watch, and heat flux (HF, W/m^2^), skin temperature (ST, °C), near-body temperature (NBT, °C), galvanic skin response (GSR, μS), and energy expenditure (EE, kcal/min) from the SenseWear armband. While the values of these variables were derived directly from experimental measurements after interpolation to ensure uniformity in the time interval of the data, the last predictor variable, 2D acceleration-mean of absolute difference (MAD, m/s^2^), was calculated from two other experimental measurements: the longitudinal acceleration-mean of absolute difference (LMAD) and transverse acceleration-mean of absolute difference (TMAD) as:

(9) $$MAD=\sqrt{TMAD^{2}+LMAD^{2}}$$

The PLS model output Y is defined as the slope of glucose change within the analyzed intervals (periods that subjects were exercising and after (up to 15–20 min depending on the last glucose measurement after completion of exercise sessions)). Glucose values measured by a glucose meter are fitted to a line and the slope of the line is used in the analysis as the Y variable. 

Before the PLS model is developed, both X and Y variables have been standardized to zero mean and unit variance. For each exercise session used in this study, the number of required latent variables, or PLS components, was determined based on the number necessary for the PLS model to describe 90% variation of X.

VIP values were calculated for each exercise session using data from all subjects. Across each exercise session, 7 AUC of biometric variables and the slope of glucose change measured by finger-sticks were calculated. Overall 149 exercise sessions are used to determine which biometric variables are the most important ones.

Table 3 shows the slopes of changes in glucose concentrations during all different exercise sessions. A slope of 0 indicates no change in glucose concentrations while negative and positive slopes indicate a decrease or increase in glucose concentrations, respectively. The more slope values deviate from 0, the more drastic increase (positive slope) or decrease (negative slope) is observed in glucose concentration values.

During six out of seven different types of exercise, glucose concentration tends to decrease. The highest mean decrease is observed during the treadmil exercise-interval training. Only during the submaximal resistance training a positive slope (increase in glucose concentration) is observed. Although in three out of five sessions a positive slope is observed, the positive mean slope obtained might be due to the small number of submaximal resistance training sessions.

Figure 1 shows the linear correlations between some of the biometric variables. Histograms of the variables appear along the matrix diagonal. Significantly high correlations between the pairs will cause repetition of the same information to be counted if multiple linear regression models are used. PLS decomposes the original data into new coordinates that are orthogonal to each other where the collinearity between the new variables is eliminated. After the collinearities are removed, multiple linear regression models are developed between the new variables and the output variable.

**Figure 2:**
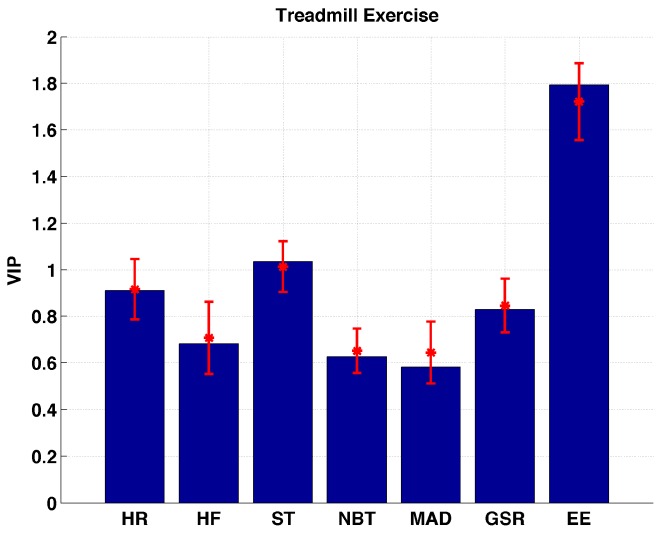
Distribution of VIP values for each biometric variable measured during treadmill exercise sessions.

Figure 2 shows the VIP values calculated for the treadmill exercise over all sessions. Bars represent VIP values calculated based on full datasets. Confidence interval for the VIP values are calculated using the Jack-knifing methodology [23]. Jack-knifing is a method for finding the precision of an estimate, by iteratively keeping out parts of the underlying data, making estimates from the subsets and comparing these estimates. The set of multiple models resulting from the cross-validation is used to calculate jack-knifing uncertainty measures (standard errors and confidence intervals). Two of the biometric variables (EE and ST) are found to be the only important variables that describe changes in glucose concentrations during treadmill exercise sessions. The VIP values for EE are found to be significantly (*p* << 0.01) higher than ST indicating that the EE is the biometric variable that can be used to describe the changes in glucose concentration induced by treadmill exercises.

**Figure 3:**
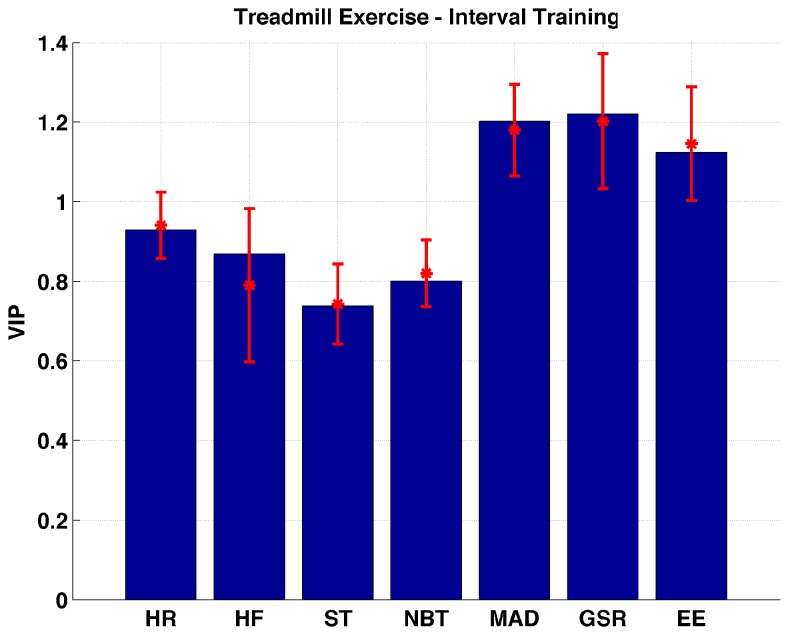
Distribution of VIP values for each biometric variable measured during treadmill exercise- interval training sessions.

During the treadmill interval exercise EE, GSR, and MAD are found to be the important variables (Figure 3). A significant difference is not observed between the importance of the three variables. The rest of the variables are found to be not important. Their importance is found to be significantly (*p* << 0.01) lower than the three important variables.

**Figure 4:**
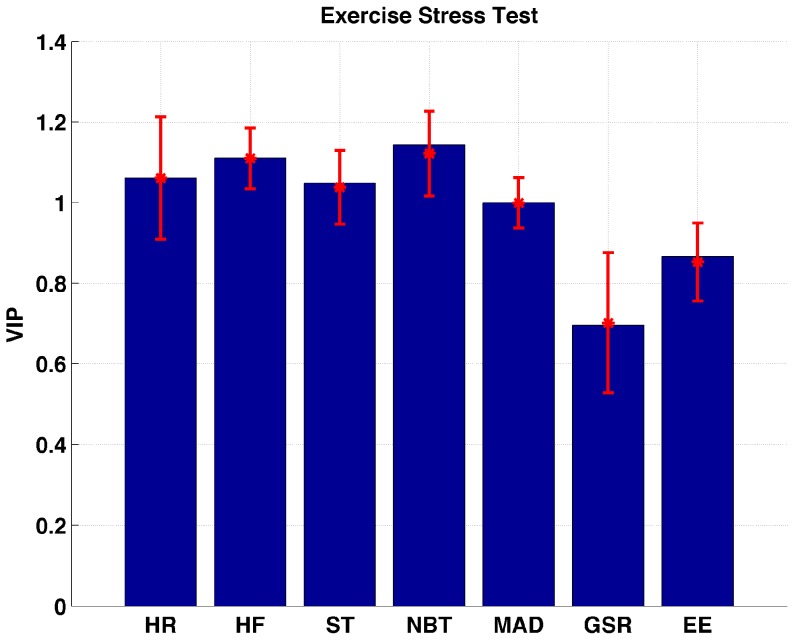
Distribution of VIP values for each biometric variable measured during exercise stress test sessions.

Figure 4 shows that all of the biometric variables except GSR and EE are found to be the important variables during and after (up to 15–20 min depending on the last finger stick after completion of exercise sessions) the exercise stress test. HF is found to be more important than ST (*p* = 0.0123) and MAD (*p* << 0.01). A significant difference between HR and other important variables is not observed. NBT correlates with the changes in glucose concentrations more than ST (*p* = 0.0133) and MAD (*p* << 0.001).

**Figure 5:**
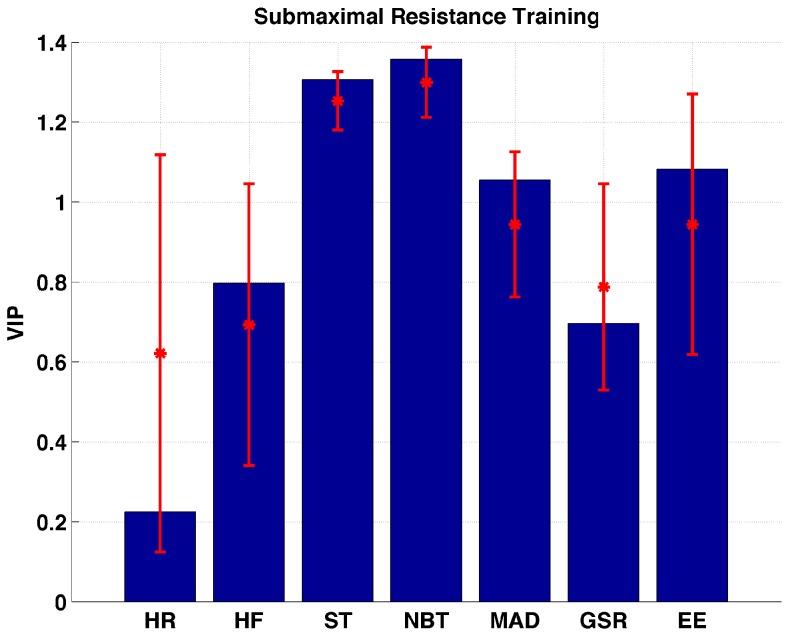
Distribution of VIP values for each biometric variable measured during submaximal resistance training sessions.

Figure 5 shows the importance of the biometric variables during submaximal resistance training. Although the HR measurements are found to be the only unimportant variable, a large variation in importance is observed when the Jack-Knife procedure is applied. HF and GSR are located within the marginally important interval. ST, NBT, MAD, and EE are the important variables to describe changes in glucose during the submaximal resistance training. A significant difference between the VIP values of ST and NBT is not observed.

**Figure 6:**
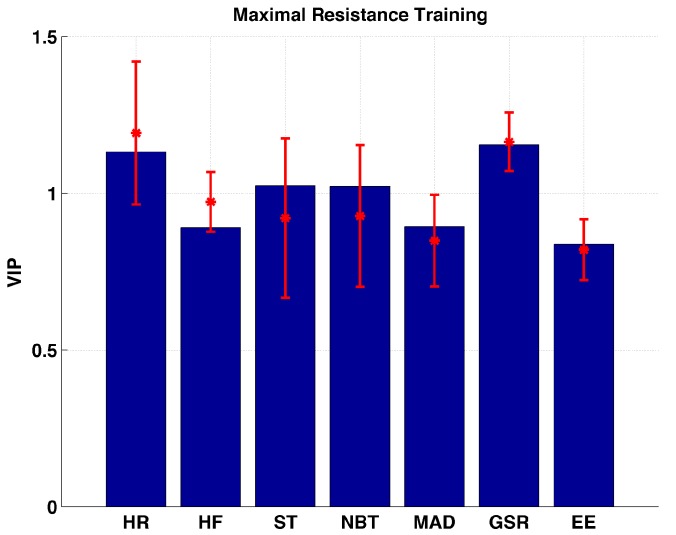
Distribution of VIP values for each biometric variable measured during maximal resistance training sessions.

HR, ST, NBT, and GSR are found to be the important variables during the maximal resistance training sessions (Figure 6). The importance of HR vs. GSR (*p* = 0.7867), and ST vs. NBT (*p* = 0.9615) are found to be almost similar. The rest of the biometric variables are located within the marginally important interval.

**Figure 7:**
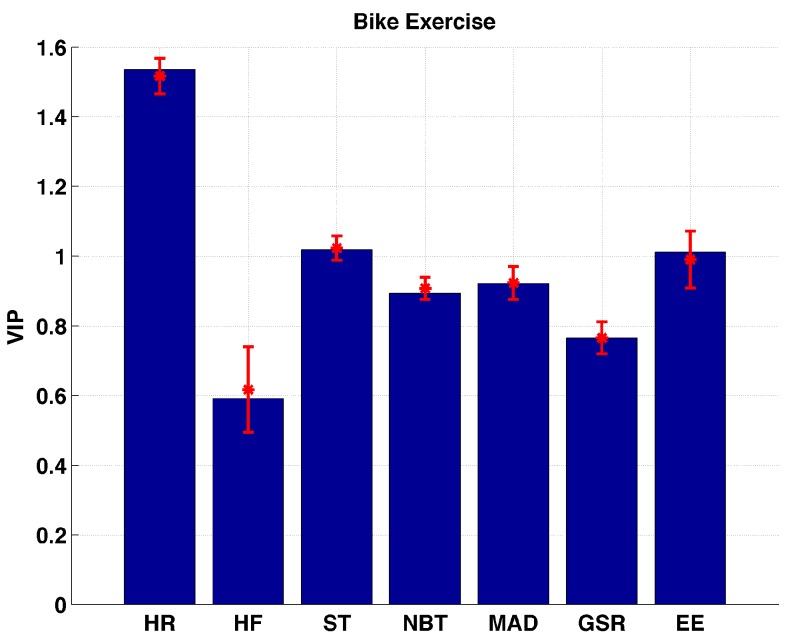
Distribution of VIP values for each biometric variable measured during bike exercise sessions.

During the bike exercise sessions HR, ST, and EE are observed to be the important variables (Figure 7). HR has the VIP value significantly (*p* << 0.01) larger than all other biometric variables. None of the variables are considered to be unimportant. The reason for HR VIP values being significantly different than all other variables is that during the bike exercises, subjects’ arms have relatively less movement, which makes the BodyMedia physical activity armband less effective at detecting the exercise. The armband is unable to track accelerometer information sufficiently.

**Figure 8:**
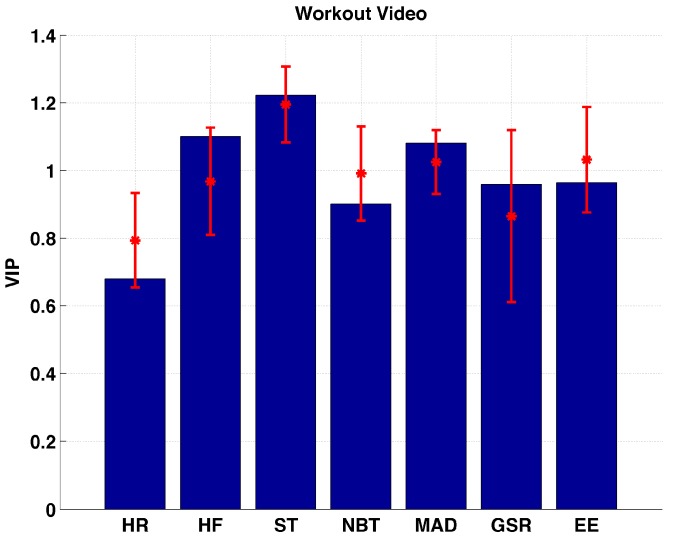
Distribution of VIP values for each biometric variable measured during workout video sessions.

Figure 8 shows that HF, ST, and MAD are found to be the important variables during the workout video sessions. None of the variables are considered to be unimportant. The VIP values for HR are found to be significantly lower (*p* < 0.0092) than all variables except GSR (*p* = 0.4032).

Overall, HR, ST, NBT, GSR, and EE are found to be the most important variables in bicycling, workout video, exercise stress test and submaximal resistance training, treadmill exercise-interval and maximal resistance training, and treadmill exercise, respectively. Over seven different types of exercises, HR, HF, ST, NBT, MAD, GSR, and EE are found to be among the important variables 3, 2, 6, 3, 3, 2, 4 times, respectively. When all of the biometric variables are sorted in descending importance order over all types of exercises and ranked (from 1 to 7 as from the least to the most important variable), an importance order (with their total ranks—maximum 49) as ST (35), EE (29), HR (29), NBT (27), GSR (26), MAD (26), HF (24) is obtained. 

Table 4 shows median (first and third quartiles) values for each the biometirc variables to interpret the directions of the correlations between the biometric variables and glucose slopes (Table 3). During all exercise sessions except submaximal resistance training, a negative slope (decrease in glucose concentration) was observed. The results in Table 3 and Table 4 indicate that the biometric variables are negatively (inversely) correlated with the glucose concentrations during exercise. When the results are further analyzed, a decreasingly negative correlation between the glucose slopes and the biometric variables is considered if the median values of the glucose slopes (note that glucose slopes have negative values) and biometric variables are closer to their first or third quartiles at the same time. Otherwise the correlation is considered to be incresaingly negative. An increasingly negative correlation indicates larger glucose decreases were observed when larger AUC of the biometric variables are calculated. A decreasingly negative correlation means, less glucose decrease was observed when larger AUC values are calculated. Out of seven biometric variables, ST and EE are found to be most consistently decreasingly negatively correlated variables with the glucose slopes. Out of 49 different variable-exercise combinations, 30 decreasingly negative correlations are observed. The decreasingly negative correlations might be because of switching from aerobic to anaerobic conditions as it is reported [24,25] that during aerobic exercise more glucose decrease is expected than during anerobic exercise conditions. 

## 4. Discussion

Most AP control systems regulate BGC in persons with T1D by using information from a continuous glucose monitor with little or no regard to physical activity levels [9,10,26,27,28,29,30,31,32,33]. Physical activity challenges the AP system as a disturbance that can lead to unsafe conditions such as hypoglycemia or hyperglycemia [34,35]. Exercise sessions are one of the most challenging periods for an AP system to regulate BGC. People with T1D have adopted a range of precautions such as modifying their insulin intake or changing their food consumption before and during exercise.

According to the American Diabetes Association, two types of physical activity are most important for managing diabetes: aerobic exercise and strength training [36]. Aerobic exercise helps the body use insulin better. It makes the heart and bones strong, relieves stress, improves blood circulation, and reduces risk for heart disease by lowering blood glucose and blood pressure and improving cholesterol levels. Cardiovascular or aerobic exercise uses glucose primarily for fuel. This means that jogging, running, the elliptical, power-walking, cycling, power yoga, anything that raises the HR for an extended period of time will lower the BGC [24,37,38]. Anaerobic activity, like strength-training, sprinting, interval, or circuit training, during which the heart rates go up and down repetitively, burns more fat for fuel during the activity and raises the BGC during the anaerobic exercise periods [25,37,38]. However, due to increase in sensitivity to insulin, reduction in BGC may be seen later than that caused by aerobic exercise periods [39,40].

We have shown that several biometric measurements such as ST, EE, and HR are highly relevant to glucose changes during different types of exercises. Among all tested variables, ST is found to be the most consistent important variable that can describe glucose changes during and after (up to 15-20 min depending on the last finger stick after completion of exercise sessions) different types of exercises.

Dehydration due to warmer temperatures may cause BGC to rise as the glucose in the bloodstream becomes more concentrated. High temperatures may also cause blood vessels to dilate, which may enhance insulin absorption, potentially leading to low blood glucose. In our experiments, we found NBT to be the most important variable during exercise stress test and submaximal resistance training, supporting the hypothesis that environment temperature may have an impact on glucose concentration. 

When the body is under stress, the adrenal glands trigger the release of glucose stored in various organs, which often leads to elevated levels of glucose in the bloodstream. The relationship between stress and GSR has already been reported [41,42,43]. We found the GSR measurements to be the most important variable during treadmill exercise–interval training and maximal resistance training sessions. In both exercise types, subjects pushed their limits, which may have caused elevated stress levels. The elevated stress level and its relation to GSR might be the reason that the GSR is found to be the most informative variable to describe the changes in glucose levels in potentially high-stress exercises.

There are other factors that can impact glycemic variability, including how much food was consumed prior to exercise, the subject’s insulin on board, and the starting glucose level prior to exercise [44]. An analysis that combines these factors and the biometric variables that we used would be an important topic to investigate. One of the limitations of our study is that all of the biometric variables have been collected under open-loop conditions. Glucose dynamics under a closed-loop system might be slightly different. Further investigation is required for the biometric variables that are collected under closed-loop conditions.

## 5. Conclusions

Overall, ST is found to be the consistently most important variable over all tested exercise types. EE and HR are also found to be important variables over many types of exercises. Although GSR and NBT are found to be important variables during some of the exercises, their importance might be related to stress or the effect of changes in environment temperature on glucose concentrations. Our results indicate that use of the additional biometric measurements in an AP system may provide better regulation of glucose concentration in patients with T1D.

## Figures and Tables

**Table 1:** Participants Characteristics (*n* = 26).

Variable	*n* (%)	Mean (SD)
Age (year)		24.2 (5.41)
Diabetes Duration (years)		12.1 (8.24)
HbA1c (%)		7.8 (1.31)
BMI (kg/m^2^)		25.1 (4.34)
Gender		
Male	12 (46.2)	
Female	14 (53.8)	
Race/Ethnicity		
White/non-Hispanic	24 (92.3)	
African American	2 (7.7)	

**Table 2:** Demographic Information (*n* = 26).

ID	Age (year)	Gender	Race/Ethnicity	BMI (kg/m^2^)	Years with Diabetes (year)	HbA1c (%)	Types of Exercise Performed
1	21.3	M	W	20.2	21.3	7.3	EST, TE, TE-I; BE; WL
2	21.3	M	W	21.0	12.9	7.4	EST, TE, TE-I; BE; WV
3	20.3	F	W	23.5	10.6	11.3	EST, TE, BE; WV
4	23.9	M	AA	24.0	16.3	8.6	EST, TE, WV
5	25.1	F	W	20.8	5.3	6.1	EST, TE, BE; WV
6	28.5	F	W	31.2	16	6.3	EST, TE, TE-I; BE; WV
7	21.8	F	W	21.4	7	8.1	TE, TE-I; BE; WV
8	34.3	M	W	23.5	31.4	8.6	EST, TE, TE-I; BE
9	22.6	F	W	27.2	11.5	8.8	EST, TE, TE-I; BE
10	22.8	M	W	31.0	10.2	6.6	EST, TE, TE-I; BE
11	19.2	F	W	24.6	7.3	9.3	EST, TE, TE-I; BE
12	32.8	F	W	38.3	29.5	7	EST, TE, TE-I; BE
13	24.9	F	W	24.0	13.7	8.2	EST, TE, TE-I; BE
14	20.8	F	W	23.6	8.8	8.7	EST, TE, TE-I; BE
15	19.5	F	W	25.7	3.3	7.2	EST, TE, TE-I; BE
16	19.4	M	W	23.3	2.5	5.1	EST, TE, TE-I; BE; WV
17	34.2	F	AA	22.1	3.2	8.4	EST, TE, TE-I; BE
18	20.7	F	W	29.2	9.8	8.2	EST, TE, TE-I; BE
19	20.2	M	W	23.8	10.4	8.3	EST, TE, TE-I; BE
20	25.3	M	W	26.5	15.3	7.1	EST, TE, TE-I; BE
21	19.5	M	W	24.6	10	9.1	EST, TE, TE-I; BE; MRT; SRT
22	19.2	M	W	22.1	7.7	8.7	EST, TE, TE-I; BE; MRT
23	22.9	M	W	21.5	6	7.4	EST, TE, TE-I; BE; MRT; SRT
24	23.2	F	W	23.0	10.3	7	EST, TE, TE-I; BE; MRT; SRT
25	39.1	M	W	33.6	31.2	7.9	EST, TE, TE-I; BE; MRT; SRT
26	27.5	F	W	22.9	2.4	5.5	EST, TE, TE-I; BE; MRT; SRT

BMI = Body Mass Index; HbA1c = Hemoglobin A1c; M = Male; F = Female; W = White; AA = African American; EST = Exercise Stress Test; TE = Treadmill Exercise; TE-I = Treadmill Exercise-Interval Training; BE = Bike Exercise; WL = Weight Lifting; WV = Exercise with Workout Video; MRT = Maximal Resistance Training; SRT = Submaximal Resistance Training.

**Table 3:** Glucose changes during different types of exercises.

Type of Exercise	Number of Sessions	Median (First, Third Quartiles) (mg/dL/min)
Treadmill Exercise	44	−1.411 (−2.33, −0.721)
Treadmill Exercise-Interval	23	−1.779 (−3.28, −0.977)
Exercise Stress Test	19	−0.311 (−1.141, 0.237)
Submaximal Resistance	5	0.245 (−0.766, 0.59)
Maximal Resistance	6	−0.257 (−0.336, −0.093)
Bike Exercise	40	−1.483 (−2.311, −0.623)
Workout Video	12	−0.41 (−0.878, −0.119)

**Table 4:** AUC (median (first–third quartiles)) of the biometric variables over all subjects during each exercise session.

Type of Exercise	HR	HF	ST	NBT	MAD	GSR	EE
TE	3948 (3470–4757)	5227 (3684–6092)	1121 (975–1462)	1087 (929–1315)	261 (212–340)	8 (5–12)	257 (212–486)
TE-I	3641 (2768–4813)	4652 (3559–6003)	1093 (932–1543)	1008 (915–1351)	259 (160–379)	5 (3–9)	246 (174–361)
EST	2344 (2169–2611)	4285 (3942–5787)	1359 (1144–1537)	1348 (1040–1498)	111 (84–123)	8 (5–11)	154 (129–260)
SRT	4186 (3008–4741)	7639 (5108–7864)	1628 (1449–1824)	1475 (1253–1610)	123 (100–158)	11 (7–23)	182 (131–282)
MRT	13633 (9236–14138)	19621 (16495–22686)	4726 (3851–5371)	4233 (3213–4810)	273 (230–368)	34 (20–79)	482 (327–708)
BE	4443 (3715–5050)	5067 (4044–6392)	1427 (1056–1581)	1313 (1011–1454)	94 (75–109)	7 (5–13)	271 (203–331)
WV	3666 (2617–4381)	5671 (3857–6654)	1289 (1116–1375)	1195 (1091–1308)	112 (92–193)	5 (4–9)	356 (297–613)
